# Interdigitating dendritic cell sarcoma presenting in the kidney combined with retroperitoneal leiomyosarcoma: A case report and literature review

**DOI:** 10.3892/ol.2013.1746

**Published:** 2013-12-09

**Authors:** JIEXIU ZHANG, BIANJIANG LIU, NINGHONG SONG, LIXIN HUA, ZENGJUN WANG, CHANGJUN YIN

**Affiliations:** Department of Urology, The First Affiliated Hospital of Nanjing Medical University, Nanjing, Jiangsu 210029, P.R. China

**Keywords:** interdigitating dendritic cell sarcoma, leiomyosarcoma, diagnosis, therapy

## Abstract

Interdigitating dendritic cell sarcoma (IDCS) and retroperitoneal leiomyosarcoma are rare tumors. The optimal diagnosis, treatment and prognosis remain unknown. The current case report presents a 46-year-old male who exhibited with a left renal mass combined with a periprostatic mass. The patient underwent surgery twice, respectively for the resection of the two masses. The postoperative pathological examination confirmed the diagnosis of IDCS presenting in the kidney and retroperitoneal leiomyosarcoma in the pelvis. To the best of our knowledge, it is the first report of IDCS in the kidney and of the combined appearance of IDCS and retroperitoneal leiomyosarcoma in the same patient.

## Introduction

Interdigitating dendritic cell sarcoma (IDCS) originates from the dendritic cell, a type of professional antigen-presenting cell that participates in innate and adaptive immune response (1). IDCS is an extremely rare tumor, which mostly occurs in the lymph node. Only a small proportion of IDCS invades extranodal sites, such as the liver, spleen, lung, intestine and bone marrow (2–5). Primary retroperitoneal leiomyosarcoma originates from retroperitoneal smooth muscle tissue, such as vascular smooth muscle, smooth muscle in retroperitoneal potential gap and residual embryonic smooth muscle (6). Retroperitoneal leiomyosarcoma is also a rare disease. The current report presents a unique case of IDCS presenting in the kidney combined with retroperitoneal leiomyosarcoma in the Department of Urology, The First Affiliated Hospital of Nanjing Medical University (Nanjing, China). Written informed consent was obtained from the patient.

## Case report

**Figure 1:**
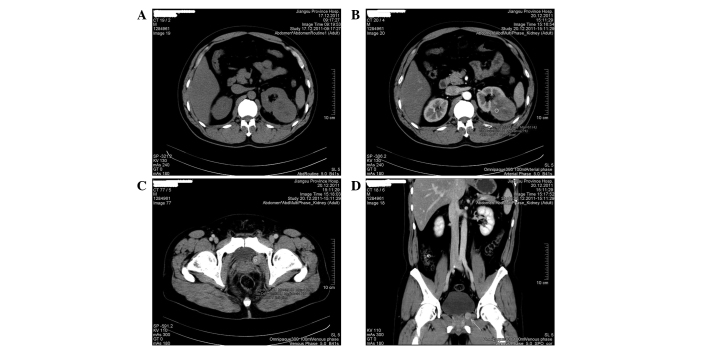
Abdominal computed tomography scan prior to the initial surgery showed (A) a roughly circular and well-demarcated mass with (B) clear enhancement effect (5.4×4.5 cm^2^ at the maximum diameter) in the lower pole of the left kidney and an additional oval-shaped mass (1.5×1.0 cm^2^ at maximum diameter) on the left side of the prostate in the (C) horizontal plane and (D) coronal plane..

A 46-year-old male was found to exhibit a left renal mass combined with a periprostatic mass by computed tomography (CT) scan during a health check in December 2011. The patient exhibited no systemic symptoms, such as fever, sweating, weight loss and fatigue. On admission, the patient's physical examination and laboratory results were normal. Abdominal CT scan showed a roughly circular, well-demarcated mass with apparent enhancement effect (5.4×4.5 cm^2^ in maximum diameter) in the lower pole of the left kidney (Fig. 1A and B). An additional 1.5×1.0-cm^2^ oval-shaped mass was identified on the left side of the prostate (Fig. 1C and D). Primary renal carcinoma and likely metastasis in the periprostatic site were considered, according to unitary theory.

**Figure 2:**
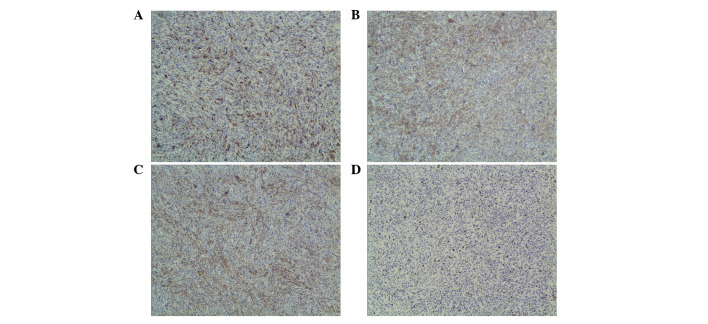
Immunohistochemistry of renal mass cells revealed positive staining for (A) S-100, (B) Vim, (C) SMA and (D) Ki67 (10% positive staining) (magnification, ×100).

**Figure 3:**
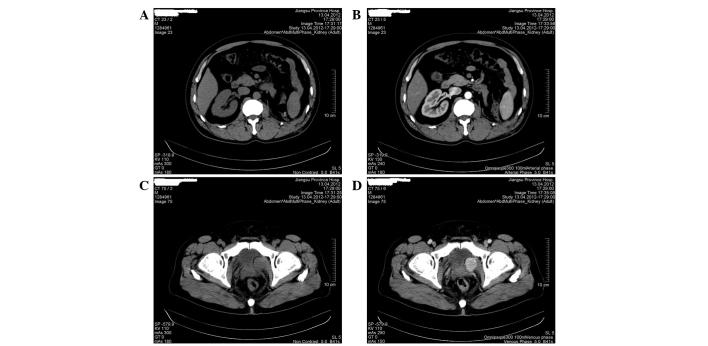
(A and B) Abdominal computed tomography scan (unenhanced) prior to the second surgery showed the absence of the left kidney without tumor recurrence or metastasis in the abdomen. (C and D) The volume of the periprostatic mass with apparent enhancement effect had evidently increased (3.5×2.8 cm^2^ at the maximum diameter) compared with that prior to the initial surgery (conrast-enhanced CT).

**Figure 4:**
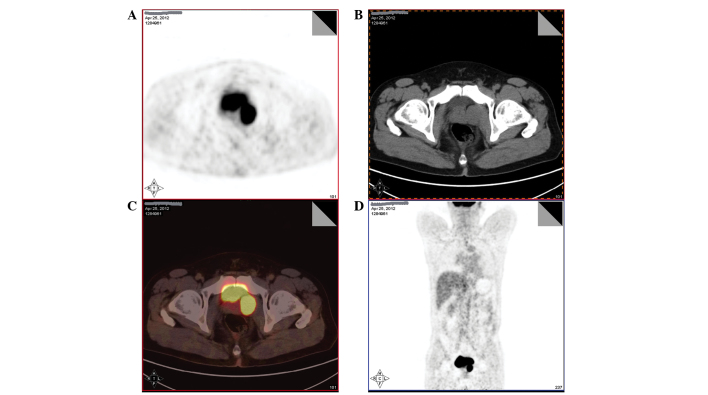
PET-CT showed a periprostatic soft tissue nodule with a 36-Hu CT value and fluorodeoxyglucose hypermetabolism without evident metastasis. (A–C) horizontal plane and (D) coronal plane. PET-CT, positron emission tomography-computed tomography.

**Figure 5:**
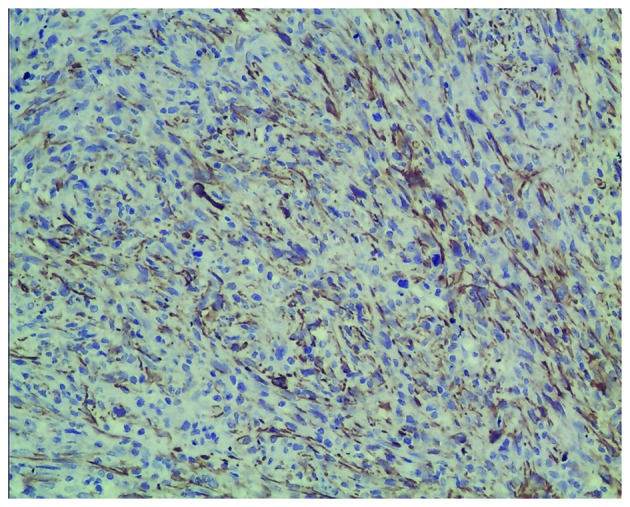
Immunohistochemistry of periprostatic mass cells showed positive staining for SMA (magnification, ×100).

**Figure 6:**
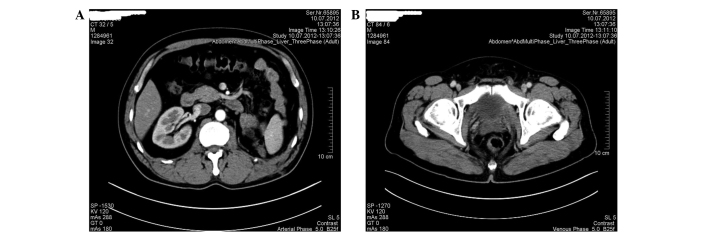
Abdominal computed tomography (CT) scan following the second surgery showed no evidence of recurrence or metastasis. (A) abdominal CT and (B) pelvic CT.

The patient underwent radical nephrectomy. During the surgery, the tumor was found to be closely adhered to the left kidney, with no definite capsule. The left kidney and mass, measuring 6×5×4 cm^3^, were completely resected. The tumor exhibited a hard texture and off-white cut surface. Microscopically, routine pathological examination suggested a malignant tumor of the left kidney. The cutting edge of the ureter, renal pelvis and renal capsule were not invaded by the tumor. The tumor had been resected completely. Immunohistochemistry showed positive staining for S-100, Vim and SMA, and negative staining for CKpan, CK7, ALK, desmin, actin, CD21, CD23, CD1a, CD34, HMB45, MelanA, RCC and CD10 (Fig. 2A–C). In total, ~10% of the tumor cells showed immunoreactivity for Ki67 (Fig. 2D). Based on the pathological results, the diagnosis of IDCS was considered. Following surgery, the patient did not receive adjuvant therapy, but received active surveillance. In April 2012, the patient underwent CT scan, which showed the absence of the left kidney without tumor recurrence or metastasis in the abdomen (Fig. 3A and B). However, the volume of the periprostatic mass had evidently increased (3.5×2.8 cm^2^ in maximum diameter), which exhibited apparent enhancement effect (Fig. 3C and D). The patient was readmitted to the Department of Urology and underwent positron emission tomography (PET)-CT to evaluate the possible metastasis. PET-CT showed a periprostatic soft tissue nodule with a 36-Hu CT value and fluorodeoxyglucose hypermetabolism, which suggested the high possibility of metastasis (Fig. 4). Therefore, the patient underwent laparoscopic pelvic tumor resection in May 2012. During surgery, the periprostatic mass was found to be located at the retroperitoneum, which had not invaded the prostate. Postoperative immunohistochemistry showed positive staining for SMA and negative staining for actin, desmin, S-100, CD21, CD23, CD117 and CD34, which demonstrated the diagnosis of retroperitoneal leiomyosarcoma (Fig. 5). Following communication with the oncologists, the patient did not receive adjuvant treatment, but received close follow-up. The patient recovered without evidence of recurrence or metastasis and the follow-up examination (abdominal CT scan only, as chest CT scan was omitted; Fig. 6) revealed no evident abnormality.

## Discussion

IDCS belongs to the dendritic cell family, which is a type of professional antigen-presenting cell, involved in innate and adaptive immune responses (1). Dendritic cell neoplasm is a rare tumor and the World Health Organization has classified dendritic cell neoplasms into the following five groups: Langerhans cell histiocytosis, Langerhans cell sarcoma, interdigitating dendritic cell sarcoma/tumor, follicular dendritic cell sarcoma/tumor and dendritic cell sarcoma (7). IDCS is exceedingly rare and, to date, <90 cases of IDCS have been reported worldwide. IDCS affects individuals of any age, with a slight male predominance, although, the majority of the cases are in middle-aged individuals (4,8). The etiology of IDCS remains obscure. A previous study have suggested that BCL2 chromosomal translocation is associated with IDCS (9), but another study contradicts this (10). The most common lesion site of IDCS is in the lymph node. In total, approximately one-third of the cases have extranodal sites, such as the liver, spleen, lung, intestine, bone marrow, nasopharynx, breast, bladder, testis and skin (2–5). To the best of our knowledge, the current case is the first reported case of extranodal IDCS in the kidney. Patients with IDCS usually present with painless lymph node enlargement or extranodal mass. Systemic symptoms, including fever, fatigue or weight loss, are extremely rare. The atypical symptoms of IDCS and its histological similarity to other soft tissue sarcomas increases the diagnostic difficulties. A correct diagnosis usually depends on postoperative pathological features (immunoreactivity for specific markers). In general, the tumor cells of IDCS are positive for S-100 and Vim and negative for CD21, CD35 and CD1a (7), which are of benefit to differentiate IDCS from other dendritic cell neoplasms. There is no standard therapeutic method for IDCS. Approximately half of the localized IDCS may be curative by successful surgery without adjuvant therapy (2,7). Systematic radiotherapy or chemotherapy is used for extensive IDCS. Chemotherapy has achieved good effects only in a few cases (11,12). However, the optimal regimen and exact role of adjuvant therapy remains unclear due to the absence of previous cases and clinical data (2,7). The reliable prognostic factors remain unknown. In general, extensive IDCS exhibits a significantly poorer prognosis than localized disease (2).

Notably, ~14.6% of patients with IDCS exhibit previous concurrent or subsequent malignancy, particularly non-Hodgkin's lymphoma (2). The current patient exhibited an additional tumor, retroperitoneal leiomyosarcoma. To date, the present report is a unique case of IDCS combined with retroperitoneal leiomyosarcoma. Primary retroperitoneal neoplasm is a rare tumor originating from retroperitoneal smooth muscle tissue, such as vascular smooth muscle, smooth muscle in the retroperitoneal potential gap and residual embryonic smooth muscle (6). The etiology of retroperitoneal leiomyosarcoma remains unknown. Rapidly increasing retroperitoneal or pelvic mass and corresponding compression symptoms are often the most common clinical manifestations. Immunohistochemistry of retroperitoneal leiomyosarcoma shows positive for SMA and desmin and negative for CD117, S-100, HMB45 and CD34, which are of benefit to differentiate leiomyosarcoma from other soft tissue tumors (13). Radical surgical resection is the main treatment (14,15). Radiotherapy or chemotherapy may be of benefit for reducing the recurrence and metastasis (14,16).

The current report presents a unique and noteworthy case. Firstly, we considered the lesion in the kidney as primary renal carcinoma and the periprostatic mass as the metastasis. The imaging study was similar to that of renal cell carcinoma. However, the final pathological examinations demonstrated IDCS and retroperitoneal leiomyosarcoma, the two distinct tumors. As far as we know, the current case of IDCS presenting in the kidney is the first to be reported. In addition, it is the first to report the combined appearance of IDCS and retroperitoneal leiomyosarcoma in the same patient. The CT and PET-CT suggested that the two tumors were localized diseases. Complete surgical resection for the masses was performed, without adjuvant therapy. Close follow-up identified no recurrence or metastasis until two-months following the final surgery. IDCS and retroperitoneal leiomyosarcoma are extremely rare tumors and optimal diagnosis, treatment and prognosis remain unknown. The study suggests that successful surgery is curative to localized IDCS and leiomyosarcoma.

## References

[1] Kadowaki N (2009). The divergence and interplay between pDC and mDC in humans. Front Biosci (Landmark Ed).

[2] Zhou J, Zhou W, Bai C, Zhou Y, Wang Y (2011). Interdigitating dendritic cell sarcoma: case report with review of the literature. Onkologie.

[3] Han HS, Lee OJ, Lim SN, An JY, Lee KM, Choe KH, Lee KH, Kim ST (2011). Extranodal interdigitating dendritic cell sarcoma presenting in the pleura: a case report. J Korean Med Sci.

[4] Ye Z, Liu F, Cao Q, Lin H (2011). Interdigitating dendritic cell sarcoma of lymph node mimicking granuloma: a case report and review of the literature. Pol J Pathol.

[5] Parada D, Peña KB, Gil I, Queralt R, Garcia A, Alos L (2012). Interdigitating dendritic cell sarcoma presenting in the nasal region. Pathol Res Pract.

[6] Shvarts O, Han KR, Lam JS, Belldegrun AS (2004). Primary leiomyosarcoma of the inferior vena cava presenting as a renal mass. Rev Urol.

[7] De Pas T, Spitaleri G, Pruneri G, Curigliano G, Noberasco C, Luini A, Andreoni B, Testori A, de Braud F (2008). Dendritic cell sarcoma: an analytic overview of the literature and presentation of original five cases. Crit Rev Oncol Hematol.

[8] Perkins SM, Shinohara ET (2013). Interdigitating and follicular dendritic cell sarcomas: a SEER analysis. Am J Clin Oncol.

[9] Nayer H, Murphy KM, Hawkins AL, Long PP, Gillison M, Borowitz M, Griffin CA (2006). Clonal cytogenetic abnormalities and BCL2 rearrangement in interdigitating dendritic cell sarcoma. Leuk Lymphoma.

[10] Wang HY, Li S, Woodford RL, Mills SE, Cousar JB (2010). BCL2 chromosomal translocation is not a general feature of the interdigitating dendritic cell sarcoma. Diagn Mol Pathol.

[11] Olnes MJ, Nicol T, Duncan M, Bohlman M, Erlich R (2002). Interdigitating dendritic cell sarcoma: a rare malignancy responsive to ABVD chemotherapy. Leuk Lymphoma.

[12] Lee SY, Lee SR, Chang WJ, Kim HS, Kim BS, Kim IS (2012). Successful treatment of disseminated interdigitating dendritic cell sarcoma with adriamycin, bleomycin, vinblastine, and dacarbazine chemotherapy. Korean J Hematol.

[13] Paal E, Miettinen M (2001). Retroperitoneal leiomyomas: a clinicopathologic and immunohistochemical study of 56 cases with a comparison to retroperitoneal leiomyosarcomas. Am J Surg Pathol.

[14] Kyriazi MA, Stafyla VK, Chatzinikolaou I, Koureas A, Chatziioannou A, Kondi-Paphiti A, Arkadopoulos N, Smyrniotis V (2010). Surgical challenges in the treatment of leiomyosarcoma of the inferior vena cava: analysis of two cases and brief review of the literature. Ann Vasc Surg.

[15] Theodosopoulos T, Psychogiou V, Yiallourou AI, Polymeneas G, Kondi-Pafiti A, Papaconstantinou I, Voros D (2012). Management of retroperitoneal sarcomas: main prognostic factors for local recurrence and survival. J BUON.

[16] Yamashita R, Muraoka K, Matsuzaki M, Matsui T, Yamaguchi R, Niwakawa M, Tobisu K, Ito I (2011). Clinicopathological study of retroperitoneal sarcoma. Nihon Hinyokika Gakkai Zasshi.

